# Risk of Buruli Ulcer and Detection of *Mycobacterium ulcerans* in Mosquitoes in Southeastern Australia

**DOI:** 10.1371/journal.pntd.0001305

**Published:** 2011-09-20

**Authors:** Caroline J. Lavender, Janet A. M. Fyfe, Joseph Azuolas, Karen Brown, Rachel N. Evans, Lyndon R. Ray, Paul D. R. Johnson

**Affiliations:** 1 Victorian Infectious Diseases Reference Laboratory, North Melbourne, Victoria, Australia; 2 WHO Collaborating Centre for Mycobacterium ulcerans</emph>, Victorian Infectious Diseases Reference Laboratory, North Melbourne, Victoria, Australia; 3 Department of Primary Industries, Attwood, Victoria, Australia; 4 Health Services Department, City of Greater Geelong, Geelong, Victoria, Australia; 5 Infectious Diseases Department, Austin Health, Heidelberg, Victoria, Australia; Faculté de Médecine, Université de la Méditerranée, France

## Abstract

**Background:**

Buruli ulcer (BU) is a destructive skin condition caused by infection with the environmental bacterium, *Mycobacterium ulcerans*. The mode of transmission of *M. ulcerans* is not completely understood, but several studies have explored the role of biting insects. In this study, we tested for an association between the detection of *M. ulcerans* in mosquitoes and the risk of BU disease in humans in an endemic area of southeastern Australia.

**Methodology/Principal Findings:**

Adult mosquitoes were trapped in seven towns on the Bellarine Peninsula in Victoria, Australia, from December 2004 to December 2009 and screened for *M. ulcerans* by real-time PCR. The number of laboratory-confirmed cases of BU in permanent residents of these towns diagnosed during the same period was tallied to determine the average cumulative incidence of BU in each location. Pearson's correlation coefficient (r) was calculated for the proportion of *M. ulcerans*-positive mosquitoes per town correlated with the incidence of BU per town. We found a strong dose-response relationship between the detection of *M. ulcerans* in mosquitoes and the risk of human disease (r, 0.99; 95% CI, 0.92–0.99; p<0.001).

**Conclusions/Significance:**

The results of this study strengthen the hypothesis that mosquitoes are involved in the transmission of *M. ulcerans* in southeastern Australia. This has implications for the development of intervention strategies to control and prevent BU.

## Author Summary

Buruli ulcer (BU) is a destructive skin condition caused by infection with the environmental bacterium, *Mycobacterium ulcerans*. BU has been reported in more than 30 countries in Africa, the Americas, Asia and the Western Pacific. How people become infected with *M. ulcerans* is not completely understood, but numerous studies have explored the role of biting insects. In 2007, it was discovered that *M. ulcerans* could be detected in association with mosquitoes trapped in one town in southeastern Australia during a large outbreak of BU. In the present study we investigated whether there was a relationship between the incidence of BU in humans in several towns and the likelihood of detecting *M. ulcerans* in mosquitoes trapped in those locations. We found a strong association between the proportion of *M. ulcerans*-positive mosquitoes and the incidence of human disease. The results of this study strengthen the hypothesis that mosquitoes are involved in the transmission of *M. ulcerans* in southeastern Australia. This has implications for the development of strategies to control and prevent BU.

## Introduction

**Figure 1:**
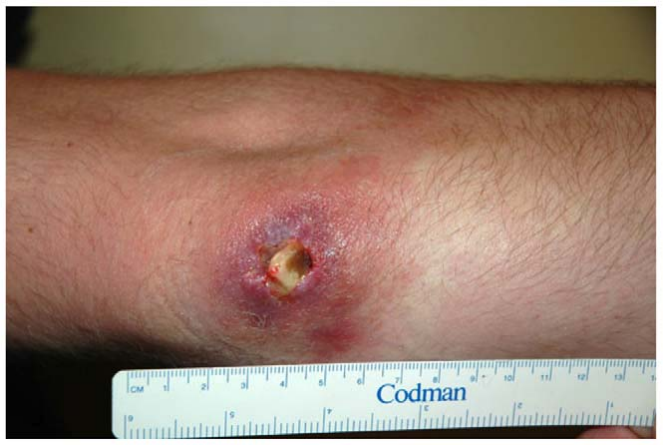
Photo of Buruli ulcer lesion. Right elbow, 32 year old male with PCR and culture confirmed Buruli ulcer. The patient spent just 4 hours at Barwon Heads on 11 May 2008 and had no known contact with any other endemic areas. He first observed the ulcer in mid-October 2008.

*Mycobacterium ulcerans* is an environmental pathogen that causes Buruli ulcer (BU), a slowly destructive infection of skin and soft tissue that can leave sufferers permanently disabled if not treated appropriately [1]. Classified by the World Health Organization (WHO) as a neglected tropical disease, BU has been reported in more than 30 countries in Africa, the Americas, Asia and the Western Pacific, mainly with tropical and subtropical climates. Australia is the only developed country with significant local transmission of BU. Foci of infection have been described in tropical Far North Queensland [2], the Capricorn Coast region of central Queensland [3], the Northern Territory [4], and temperate coastal Victoria, where it is often referred to as Bairnsdale ulcer (Figure 1) [2], [5]–[8].

*Mycobacterium ulcerans* is ancestrally related to *Mycobacterium marinum*, with which it shares >98% DNA homology, but it emerged as a separate species through the acquisition of a virulence plasmid that encodes the production of a lipid toxin, mycolactone [9], [10]. Mycolactone diffuses into tissues surrounding a focus of *M. ulcerans* infection in the dermis or subcutaneous tissue where it induces cellular apoptosis and necrosis and inhibits the local immune response [11], [12]. It is hypothesised that mycolactone confers an adaptive advantage to *M. ulcerans* by allowing it to occupy an enriched environmental niche in which some of its ancestral biosynthetic pathways are no longer required [10], [11]. However, this environmental niche, along with the mode of transmission of *M. ulcerans*, is not completely understood.

Epidemiologic investigations of BU in Australia typically show a coastal distribution and intensely localised outbreaks of disease [2], [5]–[8]. Most patients who develop BU are permanent residents of endemic areas, but others are visitors [6], [8] and a few report very brief exposure times of just hours or days (L. Brown and P.D.R. Johnson, pers. comm.). This observation, combined with incubation periods of up to seven months [13], suggest that a person's risk of acquiring BU begins immediately on entering an endemic area and that the bacterial inoculum may be quite low. One possible explanation for these clinical observations is transmission via biting insects [14]–[16].

In 2007, we first reported that *M. ulcerans* DNA could be detected in association with mosquitoes captured in a small coastal town in southeastern Australia during a large outbreak of BU [6]. However, relatively small numbers of mosquitoes collected from other locations were tested as part of that study and so it has not yet been determined if there is a quantitative relationship between the detection of *M. ulcerans* in mosquitoes and the risk of developing BU disease. In this study, we tested for an association between the detection of *M. ulcerans* DNA in mosquitoes and the incidence of BU in residents of several towns on the Bellarine Peninsula in Victoria, where most cases of BU in Australia currently occur.

## Materials and Methods

### Study site

**Figure 2:**
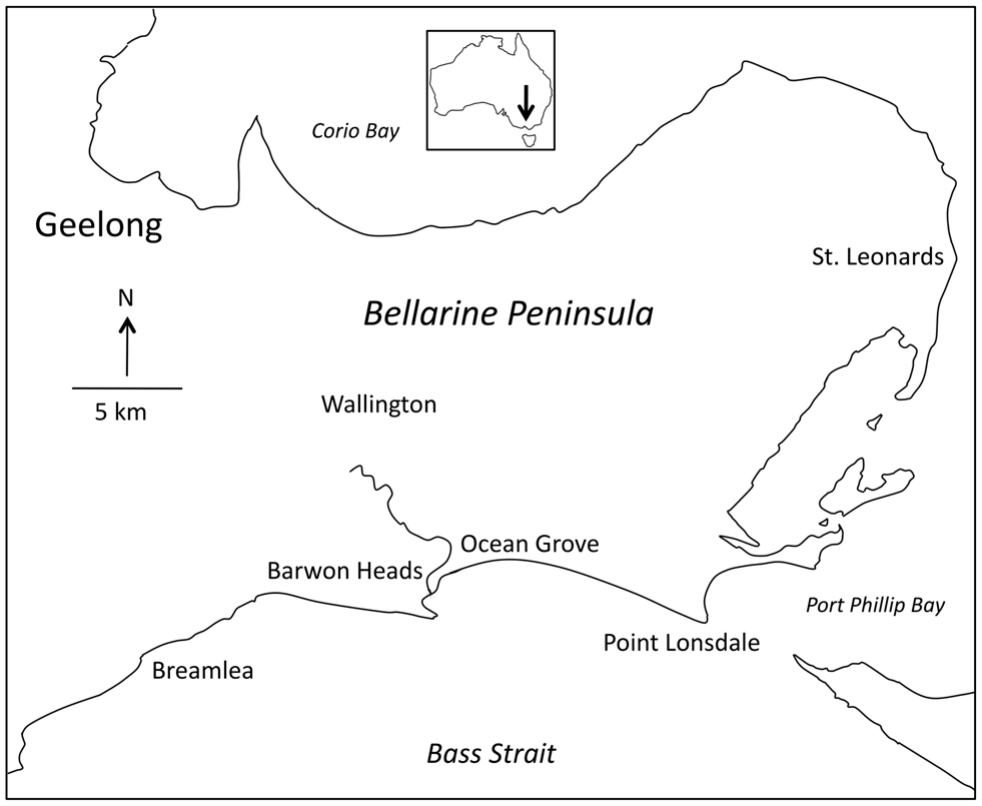
Sketch map of places and towns referred to in the text.

The Bellarine Peninsula is an area of approximately 400 square kilometres to the southwest of Melbourne, the capital of the Australian State of Victoria, which comprises a series of small towns separated by rural and coastal areas (Figure 2). The climate is temperate, with mean daily maximum temperatures ranging from 12.8°C in July (winter) to 24.5°C in February (summer) [17]. Average annual rainfall ranges from 518.5 mm in Geelong to 660.4 mm in Point Lonsdale and is spread throughout the year [17]. The Peninsula is a popular seaside holiday area and receives large numbers of visitors, particularly in summer. Accurate resident population information for towns on the Bellarine Peninsula is available from the 2006 Census of Population and Housing undertaken by the Australian Bureau of Statistics [18].

### Mosquito trapping

Adult mosquitoes were collected from December 2004 to December 2009, inclusive, using dry-ice baited miniature light traps as described previously [6]. These traps work by attracting female mosquitoes questing for a blood meal. The same trapping technique was used to collect mosquitoes in all locations, however the number of trapping nights varied in different areas. Between December 2004 and February 2006, mosquitoes were trapped monthly or every two months in Point Lonsdale by the Victorian Department of Primary Industries. From August 2006 to December 2009, mosquitoes were collected from multiple locations on the Bellarine Peninsula by the City of Greater Geelong. Traps were set fortnightly in Barwon Heads, Breamlea, Geelong, Ocean Grove, Point Lonsdale and St Leonards during the mosquito breeding season (usually August to late March). Traps were only set sporadically in Wallington. All catches were transported to the Victorian Department of Primary Industries where they were counted, sorted and pooled by sex and species as described previously [6]. Mosquito species were identified using the key of Russell (1996) [19].

### Testing mosquitoes for *M. ulcerans* by PCR

DNA was extracted from pools of ≤15 mosquitoes of the same sex and species and screened for the presence of *M. ulcerans* DNA by real-time PCR targeting three independent regions in the *M. ulcerans* genome (IS*2404*, IS*2606* and KR) as described previously [6], [20]. These assays have been validated for use on mosquitoes and other environmental samples and are able to distinguish between *M. ulcerans* and other mycolactone-producing mycobacteria that also contain IS*2404* [6], [20]–[22]. Culture was not attempted as it was neither practical/affordable (due to the low proportion of *M. ulcerans*-positive mosquitoes which would have necessitated the inoculation of thousands of samples into appropriate culture media) nor likely to be successful (due to the low number of *M. ulcerans* organisms estimated to be present on *M. ulcerans*-positive mosquitoes on the basis of real-time PCR).

### Case definition

BU was declared a notifiable disease in Victoria in 2004 and a diagnostic PCR for *M. ulcerans* is performed at one centre only (the Victorian Infectious Diseases Reference Laboratory [VIDRL]) [20]. All laboratory-confirmed cases diagnosed from 1 January 2005 to 31 December 2009, who were permanent residents of a town on the Bellarine Peninsula where mosquitoes were tested, were included in this study. A patient was considered as having acquired BU from a particular town if he/she was a resident of that town and had not reported recent contact with any other known endemic area. As accurate visitor numbers for each of the towns are not available, patients with residential addresses elsewhere, even though they may have been exposed on the Bellarine Peninsula, were classified as visitors and excluded.

### Statistical analysis

Due to the small number of BU cases in some towns, the average number of cases from each town over the five-year period was combined with population data from the 2006 Census [18] to obtain the “average cumulative incidence” by town (i.e. total number of cases per town ÷ 5 ÷ resident population of town × 1000). The proportion of *M. ulcerans*-positive mosquitoes from each town was calculated using the bias-corrected maximum likelihood estimation (MLE) method and software recommended by the Centers for Disease Control and Prevention, Atlanta [23]. This program takes into account pooling of mosquitoes during testing. Pearson's correlation coefficient (r) was calculated for the average cumulative incidence of BU per 1,000 population per town, correlated with mosquito infection rate per 1,000 mosquitoes per town, using Medicalc software (Medcalc® 11.1.1.0 for Windows; www.medcalc.be).

## Results

### Detection of *M. ulcerans* DNA in mosquitoes

A total of 41,797 mosquitoes from Barwon Heads, Breamlea, Geelong, Ocean Grove, Point Lonsdale, St Leonards and Wallington were tested for *M. ulcerans* by PCR. *Aedes camptorhynchus*, or the Southern Saltmarsh mosquito, was the predominant species caught (91% of the total catch), with an *M. ulcerans* DNA detection rate of 1.85 per 1,000 mosquitoes (95% confidence interval [CI], 1.46–2.33). The *M. ulcerans* DNA detection rate was highest in *Anopheles* sp. (10.80 per 1,000 mosquitoes; 95% CI, 0.60–54.06) and lowest in *Culex* sp. (1.02 per 1,000 mosquitoes; 95% CI, 0.06–4.91), but the wide confidence intervals make it difficult to draw meaningful conclusions about differences between mosquito species (data not shown). Negligible numbers of male mosquitoes were caught. There was a wide variation in the detection of *M. ulcerans* DNA in mosquitoes collected from different locations (Table 1). Point Lonsdale had the highest proportion of *M. ulcerans*-positive mosquitoes (4.02 per 1,000 mosquitoes; 95% CI, 3.13–5.08). *Mycobacterium ulcerans* DNA could not be detected in any mosquitoes trapped in Geelong or Wallington.

**Table 1:** Detection of *M. ulcerans* in mosquitoes, Bellarine Peninsula, 2005–09.

Location	No. mosquitoes tested	No. pools tested	No. pools PCR positive^a^	Infection rate per 1,000 mosquitoes (95% CI)^b^
Barwon Heads	4,338	366	4	0.93 (0.30–2.22)
Breamlea	3,466	274	1	0.29 (0.02–1.40)
Geelong	2,693	278	0	0 (0–1.41)
Ocean Grove	7,641	589	6	0.79 (0.32–1.64)
Point Lonsdale	16,579	1,403	69	4.27 (3.35–5.37)
St Leonards	6,200	528	1	0.16 (0.01–0.78)
Wallington	757	56	0	0 (0–4.88)
**Total**	**41,797**	**3,502**	**77**	**1.86 (1.48–2.32)**

a Positive in at least two replicates for IS*2404* (± IS*2606* and KR).

b Maximum likelihood estimate (MLE). MLE bias was corrected when ≥1 pool was positive; otherwise uncorrected.

### Incidence of BU on the Bellarine Peninsula

A total of 183 laboratory-confirmed cases of BU were notified to the Victorian Department of Health from 2005 to 2009, of which 132 (72%) were linked to the Bellarine Peninsula. Of these 132 cases, 81 were permanent residents of towns from which mosquitoes were tested and were included in the final analysis. The median age of these patients was 61 (range: 3 to 94), 58% were male and most had lesions on the lower (47%) or upper (33%) limbs. Table 2 shows the geographic variation in BU incidence on the Bellarine Peninsula during the study period. The average cumulative incidence of disease was highest in Point Lonsdale (4.04 per 1,000 population) and lowest in Breamlea and Wallington (no cases of BU identified).

**Table 2:** BU incidence per 1,000 population by town, 2005–09.

Location	Total cases	Mean no. cases per year	Resident population	Mean annual incidence (95% CI)
Barwon Heads	13	2.6	2,993	0.87 (0–1.95)
Breamlea	0	0	244	0 (0–0.012)
Geelong	5	1	160,991	0.01 (0–0.74)
Ocean Grove	10	2	11,274	0.18 (0–1.44)
Point Lonsdale	50	10	2,477	4.04 (0.81–6.4)
St Leonards	3	0.6	1,621	0.37 (0–0.43)
Wallington	0	0	1,353	0 (0–0.003)

### Association between BU incidence and detection of *M. ulcerans* DNA in mosquitoes

**Figure 3:**
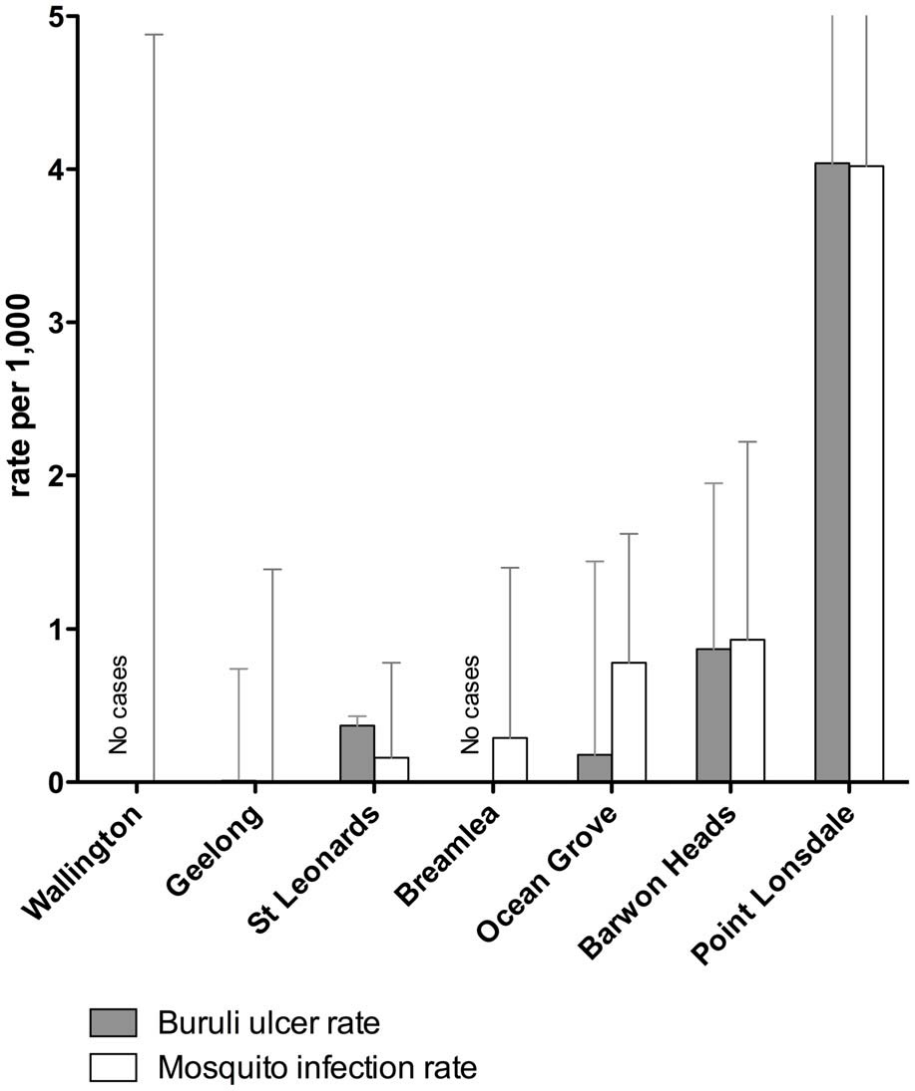
Correlation between detection of M. ulcerans in mosquitoes and incidence of BU, Bellarine Peninsula, 2005–09. The vertical axis shows the maximum likelihood estimate (MLE) of the proportion of M. ulcerans-positive mosquitoes or the average cumulative incidence of BU over five years by town (with 95% confidence intervals). There were no cases of BU at Breamlea or Wallington during the study period.

There was a positive correlation between the proportion of *M. ulcerans*-positive mosquitoes in each town and the average cumulative incidence of BU in the same location (r, 0.99; 95% CI, 0.92–0.99; p<0.001) (Figure 3). The association was strongest in Point Lonsdale and Barwon Heads, which had the highest incidences of BU and the highest proportions of *M. ulcerans*-positive mosquitoes, and in Geelong and Wallington, which had the lowest incidences of BU and the lowest proportions of *M. ulcerans*-positive mosquitoes (Figure 3). The association in St Leonards, Breamlea and Ocean Grove was not quite as striking, but the correlation was still positive within the expected statistical variation in the methods we have used.

## Discussion

BU did not occur on the Bellarine Peninsula before 1998, but since then it has become the most highly endemic area in Australia. Despite its relatively small size and the short distances between towns, there is a large geographic variation in the risk of acquiring BU. This provided an ideal opportunity to test for an association between the detection of *M. ulcerans* in mosquitoes and the risk of disease in humans. By screening large numbers of mosquitoes trapped in different towns on the Bellarine Peninsula for the presence of *M. ulcerans* DNA (Table 1), and calculating the incidence of BU in those locations (Table 2), we have identified a strong dose-response relationship between the detection of *M. ulcerans* in mosquitoes and the incidence of human disease (Figure 3). Establishing this biological gradient of risk [24] adds significantly to our previous finding that *M. ulcerans* is detectable in mosquitoes collected in one highly endemic area [6].

We acknowledge that this association does not prove causation, however it adds to the existing body of evidence implicating mosquitoes as a vector of *M. ulcerans* in Australia. In 2005, a case-control study conducted on the Bellarine Peninsula found that the use of insect repellent and being bitten by mosquitoes on the lower legs were the only two variables independently associated with BU in a multivariate analysis of risk factors [25]. In 2009, a study comparing the incidence of notifiable infectious diseases in Victoria demonstrated a statistically significant correlation between BU and the mosquito-borne diseases Barmah Forest Virus and Ross River Virus, but not between BU and other infectious diseases [26]. While not excluding alternative modes of transmission such as inoculation by direct contact, these results suggest that mosquitoes, rather than biting insects in general or exposure to the environment in general, are involved in the spread of *M. ulcerans* to humans in southeastern Australia.

Clinical observations also lend weight to the hypothesis that mosquitoes may transmit *M. ulcerans*. In most patients, the locations of lesions are in keeping with exposure to biting insects (e.g. ankles, elbows and the tip of an ear [6]). No cases have yet been described on the sole of the foot (which might be expected if the main mode of transmission was direct inoculation from a contaminated environment). There are also examples of patients who have had only very brief periods of contact with an endemic area prior to diagnosis (Figure 1) and patients who have reported that their lesion occurred at the site of a mosquito bite (L. Brown and P.D.R. Johnson, pers. comm.). Taken together, we believe that the simplest interpretation of our clinical observations, previously published data, and the results of this new study, is that mosquitoes are involved in the transmission of *M. ulcerans* in southeastern Australia [24], [27].

How mosquitoes become contaminated with *M. ulcerans*, and whether this occurs at the larval or adult stage, is unknown. However, an ongoing study on the role of mammals in the ecology of *M. ulcerans* in Australia may provide some answers [21]. One of the findings of that study was that possums (native tree-dwelling mammals) in Point Lonsdale excrete high concentrations of *M. ulcerans* DNA in their faeces. Therefore, it is possible that heavy environmental contamination of mosquito breeding sites, such as ponds, house gutters or drains, with possum faeces containing *M. ulcerans* enable mosquitoes (either as larvae or adults) to come into contact with *M. ulcerans*. The study also found that up to a quarter of captured ringtail possums in Point Lonsdale have laboratory-confirmed BU skin lesions. Thus, another possibility is that mosquitoes pick up *M. ulcerans* by feeding on a diseased possum or resting on an open wound that contains high numbers of *M. ulcerans* bacilli. Whether these contaminated mosquitoes move from one town to another is also an interesting question. Adult *Ae. camptorhynchus* mosquitoes have been noted to disperse widely from larval habitats [19], but anecdotal reports suggests that they will remain in the area if food (blood meals) is available (J. Azuolas, pers. comm.).

An alternative explanation for our observations is that the detection of *M. ulcerans* in association with mosquitoes simply reflects the presence of *M. ulcerans* in the environment and we recognise that further work is needed to demonstrate that mosquitoes are capable of transmitting *M. ulcerans*. A recent study by Wallace et al. [28] provides some insights into the potential for mosquitoes to act as biological – where an infectious agent is harboured within the mosquito and transmission is active (e.g. via a bite) – or mechanical– where a pathogen is present on the outside of the mosquito and transmission is passive (e.g. via contaminated proboscis or feet) – vectors of *M. ulcerans*. Their study found that *M. ulcerans* could be maintained throughout larval development but could not be detected in pupal or adult mosquitoes by their PCR method, suggesting that mosquitoes may not be true biological vectors of *M. ulcerans*. However, the authors also reported that the survival phenotype of *M. marinum* in *Aedes aegypti* mosquitoes was identical to that of *M. ulcerans*, which implies that the ability to produce mycolactone plays no role in assisting in the interaction between mycobacteria and insects. This contrasts with previous studies by Marsollier et al. [29] and Tobias et al. [30], which both found that wild-type *M. ulcerans* preferentially colonises insects compared with non-mycolactone producing controls. If further work resolves these conflicting findings in favour of a specific role for mycolactone in the colonisation of insects, this would provide a biologically plausible basis for our observations. Wallace at al. [28] also do not rule out the possibility of mechanical transmission of *M. ulcerans* by mosquitoes and note that if mechanical transmission were to occur it is likely that only a small number of organisms would be transferred. This scenario is consistent with our laboratory findings, which indicate that the bacterial load of PCR-positive mosquitoes is low (estimated to be 10-100 organisms per mosquito) [6], and our clinical observations that some patients with BU have long incubations periods (that could be explained by a low inoculum) [13].

There is also the question of whether mosquitoes could be involved in the transmission of *M. ulcerans* in other BU endemic areas such as Queensland, which has the second highest number of BU cases in Australia after Victoria, and Africa, where the majority of BU cases occur globally. Although there are no published data on the testing of wild-caught mosquitoes for *M. ulcerans* in these regions, several studies support a role for other biting insects [14]–[16]. For example, a study in Benin demonstrated an association between the incidence of BU in humans and the detection of *M. ulcerans* DNA in aquatic insects [1]. Although that study concerns Hemiptera and not mosquitoes, the results are in agreement with the findings of the present study. There is also epidemiological evidence from a case-control study in Cameroon that the use of bed nets is a protective factor for BU, which could implicate mosquitoes or other nocturnal biting insects as vectors [31]. In Queensland, epidemiological data and clinical observations are also consistent with a mosquito or other insect vector. A review of patients diagnosed with BU over the last 44 years revealed that most lesions occur on the exposed extremities and that some patients gave definite histories of insect bites preceding the development of their lesion [2]. The review also noted that most cases in Queensland present during the dry season and that increased numbers of cases occurred in the dry seasons that followed major flooding events [2]. Given documented incubation periods of up to seven months [13], this suggests that transmission of *M. ulcerans* in Queensland occurs during the wet season when mosquito numbers peak.

Understanding the mode of transmission of *M. ulcerans* is fundamental to the control and prevention of BU. This study has provided new evidence of a strong dose-response relationship between the detection of *M. ulcerans* in mosquitoes and the risk of disease in humans in a BU endemic area in Victoria, Australia. In the absence of an alternative theory of transmission that better explains our clinical, epidemiological and laboratory observations, we believe that mosquitoes play a role in the spread of *M. ulcerans* to humans in southeastern Australia and that public health measures to minimise mosquito bites in residents and visitors should be encouraged in endemic areas.
